# Impact of product-based e-cigarette marketing on the attitudes and behavioural intentions of young Australians: an experimental study

**DOI:** 10.1136/tc-2024-058709

**Published:** 2024-06-11

**Authors:** Michelle I Jongenelis, Kahlia McCausland, Stefan Bode, Tess Howard, Melissa Ledger, Sarah J Durkin

**Affiliations:** 1 Melbourne Centre for Behaviour Change, Melbourne School of Psychological Sciences, The University of Melbourne, Melbourne, Victoria, Australia; 2 Collaboration for Evidence, Research and Impact in Public Health, School of Public Health, Curtin University, Perth, Western Australia, Australia; 3 Minderoo Foundation, Nedlands, Western Australia, Australia; 4 Cancer Council Western Australia, Subiaco, Western Australia, Australia; 5 Centre for Behavioural Research in Cancer, Cancer Council Victoria, Melbourne, Victoria, Australia

**Keywords:** Advertising and Promotion, Electronic nicotine delivery devices, Packaging and Labelling, Prevention, Tobacco industry

## Abstract

**Background:**

The tobacco industry has a history of using language to downplay the harms associated with cigarettes and mislead consumers and policymakers. Emerging evidence suggests similar tactics are being used in the context of e-cigarettes; however, exploration of the impact of product name on attitudes towards e-cigarettes and susceptibility to use is lacking. This experimental study explored whether attitudes towards e-cigarettes and susceptibility to use are influenced by the names used by the industry to describe and market these products.

**Method:**

An accredited web panel provider recruited a sample of 383 Australians aged 12–29 years who had never smoked to participate in an online survey that featured an embedded experiment. Participants were randomly allocated to one of three conditions, each of which used a different name to describe e-cigarettes (condition 1: ‘e-cigarettes’, condition 2: ‘vapes’; condition 3: either ‘IGETS’, ‘Puff Bars’, ‘HQD Cuvies’ or ‘Gunnpods’). The survey assessed respondents’ overall opinion of the product described; attitudes towards the product; liking of the product; and curiosity, willingness and intentions to use the product.

**Results:**

Those in the ‘brand name’ condition scored higher than those in the ‘e-cigarettes’ condition on all dependent variables. Those in the ‘vapes’ condition scored higher than those in the ‘e-cigarettes’ condition on product attitude.

**Conclusion:**

Findings indicate that the use of brand names and terms such as ‘vapes’ instead of ‘e-cigarettes’ results in more favourable attitudes towards e-cigarettes and susceptibility to use among young Australians. Results highlight the problematic influence of promotional language use favoured by industry.

**WHAT IS ALREADY KNOWN ON THIS TOPIC**

- Despite emerging evidence suggesting the tobacco and e-cigarette industries are using language to downplay the harms associated with e-cigarettes and mislead consumers and policymakers, an exploration of the impact of product name on attitudes towards e-cigarettes and susceptibility to e-cigarette use is lacking.

**WHAT THIS STUDY ADDS**

- Results from this study suggest that language is important, with the use of brand names and terms such as ‘vapes’ instead of ‘e-cigarettes’ resulting in more favourable attitudes towards e-cigarettes and susceptibility to use among young Australians.

**HOW THIS STUDY MIGHT AFFECT RESEARCH, PRACTICE OR POLICY**

- To protect adolescents and young adults who have never smoked from becoming more vulnerable to e-cigarette uptake, labelling reforms should ensure that brand names cannot be used as a form of promotion.
- Those in public health should continue to use the term ‘e-cigarettes’ to appropriately convey device risk.

## Introduction

 Electronic cigarette (e-cigarette) use has increased significantly over the last decade. Since 2012, the estimated number of people using e-cigarettes has quadrupled from 21.3 to 86.1 million globally.^1^ In Australia, current use of e-cigarettes increased from 1.4% in 2018 to 8.9% in 2023, with those aged <35 years the most prevalent users of the devices.^2^ Among secondary school students, the proportion who report having ever used an e-cigarette doubled from 14% in 2017 to 29% in 2022/2023.^3^ Concerningly, increases have been observed in those who smoke and those who have never smoked,^2^ suggesting the e-cigarette market is attracting nicotine-naïve populations.

The substantial increase in e-cigarette use is likely to be partially attributable to product marketing.^4 5^ Multiple strategies are used by the tobacco and e-cigarette industries to market their products, with these strategies spanning each of the four Ps of marketing: product, place, promotion and price.^6^ Of interest to the present study is product-based marketing; in particular, the name given to the product itself. Language is powerful and words matter. They influence thought^7^ and reflect and shape understanding of health behaviours.^8 9^ The tobacco industry has a long history of using language to downplay the harms associated with cigarettes and mislead consumers and policymakers.^8^,^12^ In the context of e-cigarettes, tobacco and e-cigarette companies typically favour terms such as ‘vapes’ and ‘vapour’ in their marketing,^12^ omitting the word ‘cigarette’. This has the effect of distancing e-cigarettes from tobacco cigarettes and may result in the public associating ‘vapes’ with harmless water vapour.^8 13 14^ E-cigarette brand names serve to further obscure health risk, with research showing that a substantial minority of adolescents believe ‘Juul’ products are not e-cigarettes or do not know the nature of the products.^15^

Despite the apparent importance of language, an exploration of the impact of product name on attitudes towards e-cigarettes and susceptibility to e-cigarette use is limited. Just one study has been conducted to date, with this qualitative study of 16–40 years in the UK finding little evidence that product names play a role in product purchasing decisions.^16^ This work was limited, however, in that few outcome variables were explored, and most participants reported smoking. An empirical investigation of the impact of product-based marketing among those who have never smoked is critical to determining appeal among tobacco-free populations.

Accordingly, to inform advocacy efforts and the development of public health policies that prevent industry from using language to their advantage, the present study aimed to determine the extent to which e-cigarette marketing in the form of product name influences attitudes towards the devices and susceptibility to use among those who have never smoked tobacco cigarettes. We focused specifically on adolescents and young adults given they are the most prevalent users of e-cigarettes^2^ and health authorities have raised significant concerns about the harms associated with e-cigarette use among these population groups.^17 18^

The context of this study is Australia where a medical prescription is required to lawfully access nicotine-containing e-cigarettes and the products can only be sold through pharmacies.^19^ E-cigarettes that do not contain nicotine can be sold by retailers to those aged 18+ years in all states and territories except Western Australia.^20^ However, the tobacco industry and its retail sector allies have flouted these laws and are flooding the market with illegal products.^21^ The Federal Government is thus considering the adoption of legislation that will prohibit the retail sale of all e-cigarette products, irrespective of nicotine content, outside the pharmaceutical scheme^20^; legislation that will facilitate enforcement efforts and ensure the success of Australia’s medical model.

## Method

### Design and procedure

As part of a larger study assessing the impact of e-cigarette marketing on the attitudes and behaviours of Australians, a web-panel provider (Pureprofile) was commissioned to recruit a sample of those aged 12–29 years to participate in a national online survey that featured an embedded experiment. Quotas were set to ensure the overall sample comprised an approximately even number of men and women and those residing in low, mid and high socioeconomic status neighbourhoods. Adolescents were oversampled given significant concerns about increasing e-cigarette use in this population cohort.^17^ This study was approved by a human research ethics committee and all participants provided informed consent (for those aged <16 years, consent was also sought from their guardian/s). Pureprofile is certified by the International Organization for Standardization.

On entering the online survey, participants were asked to provide their age. Those who met the eligibility criteria (ie, were aged 12–29 years) were then asked to report their gender and postcode, after which they were randomly allocated to one of three conditions. In each of these conditions, participants were presented with a description of e-cigarettes. The descriptions were identical across conditions, except for the name given to the product being described. In condition 1, the term ‘e-cigarettes’ was used. In condition 2, the term ‘vapes’ was used. In condition 3—the ‘brand name’ condition—participants were randomly allocated to a description that featured the name of one of four popular brands of e-cigarettes in Australia: ‘IGETS’, ‘Puff Bars’, ‘HQD Cuvies’ or ‘Gunnpods’. Each of the descriptions is presented in online supplemental table S1.

After exposure to the description, participants were asked a series of questions relating to the product described. They were also asked to report on their use of e-cigarettes and tobacco cigarettes. All responses were monitored for quality, with Pureprofile automatically terminating the experiment if a participant failed more than one of the three embedded attention and logic checks.

### Measures

#### Demographics

Participants were asked to report their gender, age, postcode (used to calculate socioeconomic status as per the Australian Bureau of Statistics’ Socio-economic Index for Areas^22^), e-cigarette use and tobacco cigarette use. For e-cigarette use, participants were asked whether they had ever used an e-cigarette (even one or two puffs), with those responding in the affirmative subsequently asked on how many days over the past 30 they used e-cigarettes that (1) contained nicotine, (2) did not contain nicotine and (3) were flavoured (response options for all: 0 days, 1 or 2 days, 3–5 days, 6–9 days, 10–19 days, 20–29 days, all 30 days). These items were used to specify the following patterns of use: current use (use of e-cigarettes at least once in the last 30 days), never use (no use of e-cigarettes, not even one or two puffs) and past use (use of e-cigarettes but not in the last 30 days). To assess smoking status, participants were asked if they had ever used a tobacco cigarette (even one or two puffs). Those responding in the affirmative were asked on how many days over the past 30 they had smoked (0 days, 1 or 2 days, 3–5 days, 6–9 days, 10–19 days, 20–29 days, all 30 days). These items were used to specify the following patterns of smoking: current smoking (use of tobacco cigarettes at least once in the last 30 days), never smoking (no use of tobacco cigarettes, not even one or two puffs) and past smoking (use of tobacco cigarettes but not in the last 30 days).

#### Dependent variables

The primary dependent variables were (1) overall opinion of the product described; (2) attitudes towards the product; (3) liking of the product and (4) curiosity, willingness and intentions to use the product. For overall opinion, participants were asked ‘How would you describe your overall opinion of this product?’ (as per Jongenelis and Thoonen^23^), with responses made on a scale of 1 (very negative) to 5 (very positive). To assess attitudes, participants were presented with five 5-point semantic differential scales (as per Farrelly *et al*^24^) and asked to report whether they believed the product to be unenjoyable/enjoyable; boring/fun; stupid/smart; uncool/cool and unattractive/attractive. A grand mean comprising the semantic differential scales was computed after reliability analysis (α=0.92).

Product liking was assessed with the following item developed by the research team: ‘How much do you like the product being described?’. Responses were made on a scale of 1 (strongly dislike) to 5 (strongly like). Curiosity was assessed with the item ‘How curious are you about using the product?’ (as per Pettigrew *et al*^25^), with responses made on a scale of 1 (definitely not) to 4 (definitely yes). Intentions were assessed with the item ‘Do you intend to use the product in the next 6 months? (as per Durkin *et al*^26^), with responses made on a scale of 1 (definitely not) to 4 (definitely yes). Finally, willingness to use the product was assessed with the item ‘Suppose you were with a group of friends and they offered you the product to try. How willing would you be to try the product?’ (as per Pettigrew *et al*^25^). Responses were made on a scale of 1 (definitely not) to 4 (definitely yes).

### Statistical analysis

A randomisation check was initially conducted using analysis of variance (ANOVA) and χ^2^ analyses. Descriptive statistics were then calculated for each of the dependent variables, stratified by condition. A series of one-way ANOVAs with Fisher’s least significant difference post hoc tests was conducted to identify any significant differences in each of the dependent variables as a result of condition allocation. Finally, sensitivity analyses in the form of ANCOVAs were conducted that controlled for the use of e-cigarettes among participants. Results relating to each of the brands comprising the brand name condition are presented in online supplemental table S2. Results stratified by (1) age and (2) use of e-cigarettes are presented in online supplemental tables S3 and S4. Note that significance testing was not conducted at the level of age or e-cigarette use due to small sample sizes.

## Results

### Sample

The final recruited sample comprised 633 participants. Only those who had never smoked (n=383) were of interest to the present study as e-cigarette uptake among members of this population segment has no benefit and many harms.^27^ However, results relating to those who smoke are presented in online supplemental tables S5 and S6.

The demographic profile of participants is presented in table 1. The sample was broadly representative in terms of gender and socioeconomic status. The majority of participants reported that they had never used an e-cigarette (n=297, 78%). There were no differences identified between the samples of each condition, indicating randomisation to conditions was successful.

**Table 1:** Sample profile

Demographic	Overall		Condition 1		Condition 2		Condition 3	
			‘E-cigarettes’		‘Vapes’		‘Brand name’	
	N=383		n=120		n=130		n=133	
	N	%	N	%	N	%	N	%
Gender								
Men	190	50	56	47	63	49	71	53
Women	193	50	64	53	67	51	62	47
Age								
Mean (SD)	17.96 (4.60)		18.09 (4.80)		17.99 (4.61)		17.80 (4.44)	
Adolescents	243	63	73	61	85	65	85	64
Young adults	140	37	47	39	45	35	48	36
Socioeconomic status								
Low	120	32	36	30	38	29	46	35
Mid	169	44	57	48	52	40	60	46
High	91	24	26	22	40	31	25	19
Missing*	3	1	1	1	0	0	2	2
Vaping status								
Never use	297	78	93	77	99	76	105	79
Former use	44	11	14	12	18	14	12	9
Current use	42	11	13	11	13	10	16	12

There were no differences identified between the samples of each condition, indicating randomisation was successful.

* Excluded from calculation of proportions.

### Primary results

Descriptive statistics and one-way ANOVA results for each of the dependent variables across each of the conditions are presented in table 2 (results for those who smoke are presented in online supplemental table S5 and S6). Scores on all dependent variables were highest among those exposed to the ‘brand name’ condition. Several statistically significant differences between conditions were observed, with those in the ‘brand name’ condition scoring higher than those in the ‘e-cigarettes’ condition on all dependent variables except curiosity. Those in the ‘vapes’ condition scored higher than those in the ‘e-cigarettes’ condition on product attitude. There were no significant differences between those in the ‘brand name’ condition and those in the ‘vapes’ condition. Results from the ANCOVAs controlling for e-cigarette use were largely the same as those identified by the one-way ANOVAs (see table 2), with one additional significant difference observed: those in the ‘e-cigarettes’ condition were less likely to report intending to use the product compared with those in the ‘vapes’ condition (p=0.044).

**Table 2:** Descriptive statistics for each condition, with one-way ANOVA and Fisher’s least significant difference post hoc test results

Dependent variable	E-cigarettes n=120	Vapes n=130	Brand name* n=133	Condition main effects (unadjusted)	Condition main effects (adjusted)
	M (SD)	M (SD)	M (SD)		
Overall opinion	1.74^a^ (1.00)	1.95^ab^ (1.14)	2.16^b^ (1.19)	*F*(2,382)=4.38, p=0.013, η^2^=0.02	*F*(2,382)=4.42, p=0.013, η^2^=0.02
Attitude	1.72^a^ (0.90)	1.99^b^ (1.06)	2.08^b^ (1.12)	*F*(2,382)=3.97, p=0.020, η^2^=0.02	*F*(2,382)=4.25, p=0.015, η^2^=0.02
Liking	1.68^a^ (1.03)	1.94^ab^ (1.20)	2.00^b^ (1.22)	*F*(2,382)=2.77, p=0.064, η^2^=0.01	*F*(2,382)=2.88, p=0.057, η^2^=0.02
Curiosity	1.70^a^ (0.82)	1.79^a^ (0.95)	1.89^a^ (1.00)	*F*(2,382)=1.28, p=0.278, η^2^=0.01	*F*(2,382)=1.46, p=0.234, η^2^=0.01
Intentions	1.33^a^ (0.62)	1.51^ab^ (0.82)	1.64^b^ (0.94)	*F*(2,382)=4.76, p=0.009, η^2^=0.06	*F*(2,382)=5.56, p=0.004, η^2^=0.03
Willingness	1.63^a^ (0.79)	1.83^ab^ (0.93)	1.88^b^ (0.96)	*F*(2,382)=2.61, p=0.075, η^2^=0.04	*F*(2,382)=3.25, p=0.040, η^2^=0.02

Different superscript letters denote significant differences between conditions. These letters represent the results of the Fisher’s LSD post hoc tests.

* Participants were exposed to one of the following brand names: IGETS, Puff Bars, HQD Cuvies or Gunnpods. These brands were chosen because of their popularity in Australia.

ANOVA, analysis of variance.

## Discussion

Despite the importance of ensuring that terminology relating to e-cigarettes serves public health interests and not the interests of tobacco companies,^28^ research assessing the impact of product naming is lacking. This experimental study sought to explore whether attitudes towards e-cigarettes and susceptibility to use are influenced by the names used by industry to describe and market these products.

The results of this study indicate that e-cigarette brand names have a promotional effect when compared with generic product descriptions of ‘e-cigarette’. Exposure to brand names such as IGET and Gunnpods was more likely than exposure to the term ‘e-cigarettes’ to result in favourable attitudes to e-cigarettes, greater liking of the product and susceptibility to use. Exposure to the term ‘vapes’ was also more likely than exposure to the term ‘e-cigarettes’ to result in favourable attitudes to e-cigarettes and susceptibility to use. These results are consistent with the impact of product-based marketing on attitudes and behavioural intentions in the tobacco control space,^8 9^ highlighting the problematic influence of promotional language use favoured by industry. To prevent the tobacco and e-cigarette industries from using language to their advantage, it is recommended that legislation be passed regulating product-based marketing to ensure brand names or terms such as ‘vapes’ cannot be used when communicating with consumers. It is also important that those in public health continue to use the term ‘e-cigarettes’ to appropriately convey device risk,^14^ bearing in mind that mass media campaigns should use language that is understood by the target audience.

These findings contrast with one prior qualitative study conducted in the UK that found product names did not play a role in product purchasing decisions.^16^ There were several key methodological differences between the present study and this prior study that likely contributed to the differing results. Given the qualitative methodology in the previous study, participants were directly asked about the influence of product packaging and product names on their purchasing decisions. This direct line of questioning assumes that participants are consciously aware of product-label-based marketing and the possible influence it may have on them, and are willing to disclose that they are influenced by this form of marketing. By contrast, the experimental methodology adopted in the present study provides a more objective method for examining the influence of this product-label-based marketing.

There are two key limitations to the present study. First, we assessed behavioural intentions rather than actual behaviour. Given the intentions-behaviour gap,^29^ future research could consider assessing the impact of product names on actual e-cigarette use. Second, inferential analyses examining the influence of age were not able to be conducted given the small sample size when stratifying participants at this level. The sample size also precluded analyses exploring differences between individual brand names, although results suggest the term ‘HQD Cuvies’ may be more appealing than ‘Puff Bars’, ‘IGETS’ and ‘Gunnpods’. Research that explores the characteristics of brand names that increase the appeal of e-cigarettes would assist with building the evidence base needed to inform labelling reforms that seek to restrict the use of promotional brand names and variants.

### Implications

With e-cigarette products currently the subject of extensive reforms in Australia, there are opportunities for this study to inform the drafting of legislative amendments. The previous Tobacco Advertising Prohibition Act 1992 (Cth) and Plain Packaging Act 2011 (Cth) allowed the use of brand and variant names as one of the very last remaining forms of tobacco promotions. Recognising this, the Public Health (Tobacco and Other Products) Act 2023 (Cth) imposes new restrictions on brand and variant names for tobacco products. Terms or features that (1) imply no or reduced harm; (2) refer to positive health effects or prohibited ingredients and (3) refer to a positive quality, a colour, filters, non-alphabetical characters, numerals or emojis are prohibited under the Act. This new legislation only broadly prohibits e-cigarette advertisements, meaning that e-cigarette brand names could be used as a form of promotion. As was the case with tobacco products, it can be expected that e-cigarette manufacturers will manipulate brand and variant names for maximum appeal. Findings underscore the need for explicit restrictions on e-cigarette brand names and variants on package labels, as well as in any advertising to health professionals that is permitted for prescription medicines. These restrictions could form part of the Australian Government’s plans to strengthen the Therapeutic Goods Order 2021, which sets out product requirements for therapeutic vaping products.

## Conclusion

Findings from the present study indicate that the use of brand names and terms such as ‘vapes’ instead of ‘e-cigarettes’ results in more favourable attitudes towards e-cigarettes and susceptibility to use. To protect adolescents and young adults who have never smoked from becoming more vulnerable to e-cigarette uptake, labelling reforms should ensure that brand names cannot be used as a form of promotion.

## Supplementary material

10.1136/tc-2024-058709 online supplemental file 1

## Data Availability

Data are available on reasonable request.
