# Supplementary material for: Impact of product-based e-cigarette marketing on the attitudes and behavioural intentions of young Australians: an experimental study
Source: Tob Control. 2024 Jun 11;34(5):e058709. doi: 10.1136/tc-2024-058709 (PMC12573368; doi:10.1136/tc-2024-058709)
Supplement: online supplemental file 1 [file tc-34-5-s001.docx]

**Supplementary Material**

Table S1. *Condition descriptions*

| Condition | Text |
| --- | --- |
| 1 | ***E-cigarettes*** are battery-powered devices that people use to heat up a liquid that usually contains flavourings and other chemicals. This e-liquid might also contain nicotine. Once heated, an aerosol is produced that users then inhale. |
| 2 | ***Vapes*** are battery-powered devices that people use to heat up a liquid that usually contains flavourings and other chemicals. This e-liquid might also contain nicotine. Once heated, an aerosol is produced that users then inhale. |
| 3 | ***Puff Bars*** are battery-powered devices that people use to heat up a liquid that usually contains flavourings and other chemicals. This e-liquid might also contain nicotine. Once heated, an aerosol is produced that users then inhale.  OR  ***HQD Cuvies*** are battery-powered devices that people use to heat up a liquid that usually contains flavourings and other chemicals. This e-liquid might also contain nicotine. Once heated, an aerosol is produced that users then inhale.  OR  ***IGETs*** are battery-powered devices that people use to heat up a liquid that usually contains flavourings and other chemicals. This e-liquid might also contain nicotine. Once heated, an aerosol is produced that users then inhale.  OR  ***Gunnpods*** are battery-powered devices that people use to heat up a liquid that usually contains flavourings and other chemicals. This e-liquid might also contain nicotine. Once heated, an aerosol is produced that users then inhale. |

Table S2. *Descriptive statistics for each brand name condition*

| Dependent variable | Puff Bars  *n* = 34 | HQD Cuvie  *n* = 28 | IGET  *n* = 34 | Gunnpod  *n* = 37 |
| --- | --- | --- | --- | --- |
| Overall opinion | 2.18 (1.24) | 2.54 (1.26) | 1.76 (0.78) | 2.22 (1.34) |
| Attitude | 1.92 (1.10) | 2.64 (1.20) | 1.94 (0.98) | 1.93 (1.11) |
| Liking | 2.03 (1.22) | 2.29 (1.33) | 1.88 (1.12) | 1.86 (1.23) |
| Curiosity | 1.82 (0.94) | 2.14 (1.04) | 1.71 (0.91) | 1.92 (1.09) |
| Intentions | 1.50 (0.83) | 2.04 (1.17) | 1.53 (0.83) | 1.57 (0.90) |
| Willingness | 1.79 (0.95) | 2.04 (1.04) | 1.76 (0.89) | 1.95 (1.00) |

*Note*. Significance testing not conducted due to small sample sizes.

Table S3. *Descriptive statistics for each condition, stratified by age*

| Dependent variable | E-cigarettes  *N* = 120 | | | | Vapes  *N* = 130 | | | Brand name  *N* = 133 | | | |
| --- | --- | --- | --- | --- | --- | --- | --- | --- | --- | --- | --- |
|  | Adolescents | Young adults | Adults 25+ | Adolescents | | Young adults | Adults 25+ | Adolescents | Young adults | Adults 25+ |  |
|  | *n* = 73 | *n* = 31 | *n* = 16 | *n* = 85 | | *n* = 28 | *n* = 17 | *n* = 85 | *n* = 31 | *n* = 17 |  |
| Overall opinion | 1.75 (0.93) | 1.55 (0.77) | 2.06 (1.57) | 2.04 (1.16) | | 1.68 (1.09) | 2.00 (1.12) | 2.09 (1.16) | 2.06 (1.21) | 2.65 (1.27) |  |
| Attitude | 1.66 (0.84) | 1.76 (0.87) | 1.93 (1.24) | 2.03 (1.12) | | 1.74 (0.91) | 2.15 (0.98) | 1.99 (1.13) | 2.19 (0.92) | 2.34 (1.38) |  |
| Liking | 1.59 (0.96) | 1.74 (1.03) | 1.94 (1.34) | 2.02 (1.25) | | 1.36 (0.73) | 2.47 (1.23) | 2.01 (1.19) | 1.74 (1.03) | 2.41 (1.58) |  |
| Curiosity | 1.70 (0.85) | 1.74 (0.77) | 1.62 (0.81) | 1.92 (1.00) | | 1.36 (0.62) | 1.88 (0.99) | 1.95 (0.99) | 1.61 (0.84) | 2.06 (1.25) |  |
| Intentions | 1.32 (0.64) | 1.35 (0.61) | 1.31 (0.60) | 1.61 (0.90) | | 1.25 (0.59) | 1.41 (0.62) | 1.69 (0.96) | 1.39 (0.76) | 1.82 (1.07) |  |
| Willingness | 1.63 (0.79) | 1.68 (0.83) | 1.56 (0.73) | 1.94 (0.96) | | 1.43 (0.69) | 1.94 (1.03) | 1.91 (0.96) | 1.61 (0.80) | 2.24 (1.15) |  |

*Note*. Significance testing not conducted due to small sample sizes.

Table S4. *Descriptive statistics for each condition, stratified by use of e-cigarettes*

| Dependent variable | E-cigarettes  *N* = 120 | | | Vapes  *N* = 130 | | | Brand name  *N* = 133 | | |
| --- | --- | --- | --- | --- | --- | --- | --- | --- | --- |
|  | Current use | Past use | Never use | Current use | Past use | Never use | Current use | Past use | Never use |
|  | *n* = 13 | *n* = 14 | *n* = 93 | *n* = 13 | *n* = 18 | *n* = 99 | *n* = 16 | *n* = 12 | *n* = 105 |
| Overall opinion | 2.00 (1.00) | 2.07 (1.44) | 1.66 (0.92) | 3.23 (0.93) | 1.78 (0.73) | 1.82 (1.13) | 2.75 (1.06) | 2.00 (1.21) | 2.09 (1.19) |
| Attitude | 1.94 (0.78) | 2.27 (1.14) | 1.61 (0.85) | 3.11 (0.92) | 2.02 (0.98) | 1.83 (1.01) | 2.81 (1.06) | 2.37 (0.63) | 1.94 (1.13) |
| Liking | 1.92 (1.19) | 1.93 (1.44) | 1.60 (0.93) | 3.31 (1.18) | 2.11 (1.18) | 1.73 (1.09) | 2.75 (1.13) | 1.75 (0.97) | 1.91 (1.23) |
| Curiosity | 2.31 (0.95) | 2.14 (0.86) | 1.55 (0.73) | 3.00 (0.58) | 2.00 (0.97) | 1.60 (0.87) | 2.94 (0.85) | 1.92 (0.90) | 1.72 (0.94) |
| Intentions | 1.85 (0.99) | 1.50 (0.65) | 1.23 (0.51) | 2.77 (0.60) | 1.56 (0.70) | 1.33 (0.71) | 2.75 (0.77) | 1.58 (1.00) | 1.48 (0.84) |
| Willingness | 2.23 (1.01) | 2.14 (0.86) | 1.47 (0.67) | 2.92 (0.64) | 2.56 (0.98) | 1.56 (0.77) | 3.00 (0.82) | 1.92 (0.79) | 1.70 (0.89) |

*Note*. Significance testing not conducted due to small sample sizes.

Table S5. *Descriptive statistics for each condition among those who smoke*

| Dependent variable | E-cigarettes  *n* = 34 | Vapes  *n* = 29 | Brand name^±^  *n* = 31 |
| --- | --- | --- | --- |
|  | M (SD) | M (SD) | M (SD) |
| Overall opinion | 3.35 (1.25) | 3.52 (0.99) | 3.23 (1.20) |
| Attitude | 3.40 (1.00) | 3.46 (1.07) | 3.01 (1.38) |
| Liking | 3.59 (1.13) | 3.45 (1.06) | 3.52 (1.21) |
| Curiosity | 2.94 (0.81) | 2.97 (0.87) | 2.71 (0.97) |
| Intentions | 3.00 (0.89) | 2.79 (0.82) | 2.35 (1.05) |
| Willingness | 3.24 (0.65) | 3.10 (1.01) | 3.00 (1.00) |

*Note*. Significance testing not conducted due to small sample sizes.

^±^Participants were exposed to one of the following brand names: IGETS, Puff Bars, HQD Cuvies, or Gunnpods. These brands were chosen because of their popularity in Australia.

Table S6. *Descriptive statistics for each brand name condition among those who smoke*

| Dependent variable | Puff Bars  *n* = 7 | HQD Cuvie  *n* = 10 | IGET  *n* = 7 | Gunnpod  *n* = 7 |
| --- | --- | --- | --- | --- |
| Overall opinion | 3.14 (1.57) | 3.80 (0.92) | 3.29 (0.76) | 2.43 (1.27) |
| Attitude | 3.17 (1.47) | 3.24 (1.45) | 3.40 (1.23) | 2.14 (1.23) |
| Liking | 3.57 (1.51) | 4.00 (1.16) | 3.71 (0.49) | 2.57 (1.13) |
| Curiosity | 2.57 (0.98) | 2.80 (1.03) | 3.00 (1.00) | 2.43 (0.98) |
| Intentions | 2.14 (1.22) | 2.60 (0.97) | 2.71 (0.76) | 1.86 (1.22) |
| Willingness | 2.71 (1.38) | 3.20 (1.03) | 2.86 (0.90) | 3.14 (0.69) |

*Note*. Significance testing not conducted due to small sample sizes.
